# Examining the associations between mental well-being, emotional regulation, social anxiety, and excessive smartphone use

**DOI:** 10.1192/j.eurpsy.2025.940

**Published:** 2025-08-26

**Authors:** S. Csibi, N. Pirwani, M. Csibi, A. Szabo

**Affiliations:** 1 Faculty of Psychology, Department of Sciences and Letters, George Emil Palade University of Medicine, Pharmacy, Science, and Technology, Targu Mures, Romania; 2 Institute of Health Promotion and Sport Sciences, Faculty of Education and Psychology, ELTE Eötvös Loránd University, Budapest; 3 Institute of Special Education, Faculty of Pedagogy, Eszterházy Károly Catholic University, Eger, Hungary

## Abstract

**Introduction:**

Interpreting and perceiving adequately others’ emotions and the regulative processes within one’s mental health are barriers or predisposing factors in the development of smartphone addiction.

**Objectives:**

The research explores the role of mental health, fear of negative perception, and assessment of other people’s emotions, which influence excessive smartphone use.

**Methods:**

The survey included 400 respondents, of whom 104 were men (26%), 293 women (73.2%), and three persons (0.8%) who indicated a different gender. The mean age of the participants was 25.9 years (SD 10.9). Registered answers refer to demographic data (gender, age, smartphone usage habits) as well as psychological measures: a Smartphone Application-Based Addiction Scale (SABAS), a Mental Health Continuum Scale (MHC), an Assessing Emotions Scale (AES), and Fear of Negative Perception Questionnaire (FNPQ).

**Results:**

Results show a significant negative correlation between the SABAS score and global mental well-being (*r*(398) = -.15, *p* = .005) and a significant positive correlation between the SABAS score and fear of negative perception (*r*(398) = .27, *p* = .001). Using SABAS’s cutoff point (23 points), non-problem (*M* = 59.6, *SD* = 11.4) and problem users (*M* = 55.8, *SD* = 11.3) differ significantly in global mental well-being (*t*(398) = -2.9, *p* = .004) and each of its sub-factors, emotional, social, and psychological well-being; as well as non-problem (*M* = 20, *SD* = 8.2) and problem users (*M* = 24.1, *SD* = 8.4) in fear of negative perception (*t*(398) = 4.3, *p* = .001). Relevant associations between emotional regulation and problematic smartphone use we did not find.

**Conclusions:**

The resulting data will support to investigation of the role of mental health well-being factors in the development of problematic smartphone usage, besides prevention and psychotherapeutic intervention.

**Disclosure of Interest:**

None Declared

